# Recent advances in the potential role of RNA N4-acetylcytidine in cancer progression

**DOI:** 10.1186/s12964-023-01417-5

**Published:** 2024-01-17

**Authors:** Shujun Zhang, Yafeng Liu, Xiao Ma, Xiaohui Gao, Yi Ru, Xinjun Hu, Xinyu Gu

**Affiliations:** 1 Department of Infectious Diseases, The First Affiliated Hospital, College of Clinical Medicine, Henan University of Science and Technology, Luoyang, 471000 Henan China; 2 Department of Oncology, The First Affiliated Hospital, College of Clinical Medicine, Henan University of Science and Technology, Luoyang, 471000 Henan China; 3 Hepatobiliary Pancreatic Surgery, The First Affiliated Hospital, College of Clinical Medicine, Henan University of Science and Technology, Luoyang, 471000 Henan China; 4 Department of Orthopedics, The Second Affiliated Hospital of Zhejiang University School of Medicine, Hangzhou, China

**Keywords:** N-acetyltransferase 10, N4-acetylcytidine, Tumor, mRNA

## Abstract

N^4^-acetylcytidine (ac^4^C) is a highly conserved chemical modification widely found in eukaryotic and prokaryotic RNA, such as tRNA, rRNA, and mRNA. This modification is significantly associated with various human diseases, especially cancer, and its formation depends on the catalytic activity of N-acetyltransferase 10 (NAT10), the only known protein that produces ac^4^C. This review discusses the detection techniques and regulatory mechanisms of ac^4^C and summarizes ac^4^C correlation with tumor occurrence, development, prognosis, and drug therapy. It also comments on a new biomarker for early tumor diagnosis and prognosis prediction and a new target for tumor therapy.

Video Abstract

**Supplementary Information:**

The online version contains supplementary material available at 10.1186/s12964-023-01417-5.

## Introduction

The connotation of classical genetics is the change of gene function caused by the change of gene sequence, which leads to the heritable change of phenotype [1]. Epigenetics, on the other hand, refers to heritable changes in gene function that ultimately lead to phenotypic changes when the DNA sequence of a gene is unchanged [1, 2]. Epigenetics is crucial in the development of multiple diseases, influencing RNA structure and function through post-transcriptional modifications [3]. They modify DNA, RNA, or histones and involve various events, such as methylation, acetylation, lactation, and glycosylation [4].

The recent development of second-generation sequencing has put RNA modifications at the forefront of genomics, allowing them to become research hotspots. These modifications are epigenetic and encompass the addition, deletion, or change of chemical groups in RNAs and further modifications of their chemical structure. Hence, chemical RNA modifications represent a new mechanism for post-transcriptional regulation of gene expression [5]. Three different types of proteins mediate RNA epigenetic modifications and change the RNA fate by writing (catalyzing the formation of RNA modifications), erasing (removing RNA modifications), and reading (identifying and binding RNA modification sites) [6]. So far, more than 100 chemically modified nucleotides have been discovered [7], with methylation and acetylation being the most common and studied type of RNA modification. Common methylation modifications include 5-methylcytosine (m^5^C), N^1^-methyladenosine (m^1^A), N^6^-methyladenosine (m^6^A), 7-methylguanosine (m^7^G), and pseudouracil (ψ), while acetylation modifications include N^4^-acetyl-2′-O-methylcytidine (ac^4^Cm), N^6^-acetyladenosine (ac^6^C), and N^4^-acetylcytidine (ac^4^C) [5, 8, 9].

Among them, ac^4^C is a ubiquitous, highly conserved chemical modification [10, 11] found in most eukaryotic and prokaryotic RNAs, including tRNAs, rRNAs, and mRNAs. It is related to mRNA stability maintenance and was first identified on tRNAs and rRNAs and later on numerous mRNAs [12, 13]. ac^4^C modification can increase gene expression by maintaining mRNA stability and improving translation efficiency.N-acetyltransferase 10 (NAT10) protein is the only known ac^4^C writer protein [12, 14] and produces ac^4^C residues on multiple RNA sites [15]. It regulates mRNA stability and translation efficiency by catalyzing the formation of ac4C modification and is involved in numerous cellular processes in living organisms, such as cell death, including apoptosis and autophagy, through its acetyltransferase activity [16]. Cancer is the leading life-threatening disease that can be improved with surgery, radiotherapy, and chemotherapy. However, patients with cancer are often faced with poor survival prognosis since the disease is frequently diagnosed at later stages. Therefore, finding new ways for early cancer diagnosis is crucial for improving the therapeutic outcome. Interestingly, the occurrence and development of various cancers have been recently associated with NAT10. The mechanism is that NAT10 catalyzes the formation of ac4C modification in mRNA, maintains mRNA stability and improves translation efficiency, thereby increasing gene expression and thus promoting tumor formation.

In the following sections, we will summarize the methods for detecting ac^4^C modification and its regulatory mechanisms. Next, we will discuss ac^4^C roles in the progression of various tumors and describe a new biomarker for the early diagnosis of tumors and a new target for tumor therapy.

### Regulatory mechanisms of ac^4^C

Since ac^4^C is a newly identified RNA modification, the mechanisms of ac^4^C formation and action are still unexplored. So far, only one writer protein of ac^4^C has been found, while eraser and reader proteins remain unknown [8]. The mechanism of the only known ac^4^C writer protein NAT10 that promotes the formation of ac^4^C residues in RNA remains to be studied.

#### Regulatory factors

Reader and eraser proteins that regulate ac^4^C in RNA have not yet been discovered [8, 17]. The ac^4^C writer protein NAT10 was first reported in 2003 and discovered to possess histone acetylation activity [18]. It is an RNA acetyltransferase that catalyzes the formation of ATP-dependent acetylation in RNA and is the only known ac^4^C writer protein [14]. It belongs to the N-acetyltransferases from the G protein subunit alpha transducin (GNAT) superfamily that catalyze acetylation on histone and non-histone proteins [19–21]. The NAT10 protein contains an acetylase domain and an RNA-binding domain [12] that allow it to catalyze ac^4^C formation in various transcripts, such as 18S rRNA, tRNA, and mRNA [8, 14]. The catalysis consumes acetyl-CoA and ATP [22, 23], and in some instances, such as during ac^4^C formation in tRNA, the assistance of THUMPD1 adaptor protein [23–25]. Moreover, ac^4^C formation also requires binding the antisense sequence of snoRNA to the target sequence [22, 26]. Interestingly, the cofactors necessary for ac^4^C formation in mRNA have not been found, and whether ac^4^C sites can be deacetylated in various RNAs remains unknown [22].

#### Location of ac^4^C sites and their effects on RNA

**Fig. 1:**
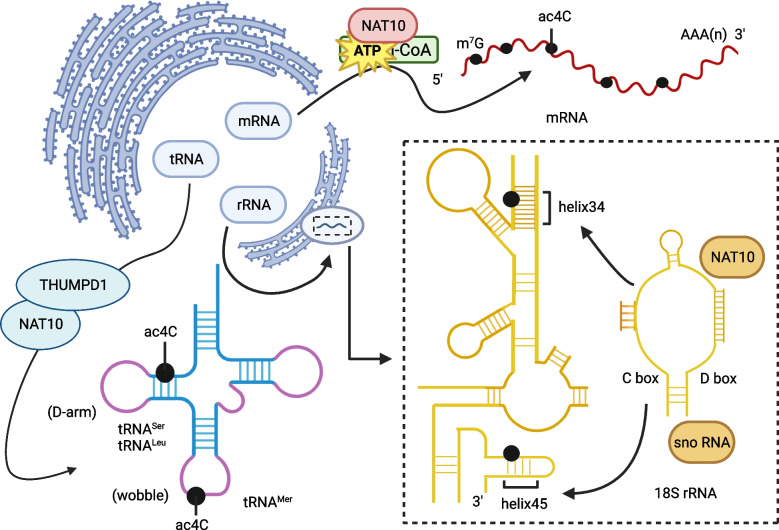
Localization sites of ac4C modification in mRNA, tRNA, and rRNA

N^4^-acetylcytidine modification is abundant in RNA and is enriched in tRNA, mRNA, and rRNA (Fig. 1). It was first identified in yeast tRNA in 1966 [22] and in rRNA in 1978 [27]. This modification is found at the wobble base of tRNA^Met^ and the D-arm of tRNA^Ser^ and tRNA^Leu^ [25, 28]. The NAT10 writer protein mediates ac^4^C modification at nucleotides 1842 and 1337 of mammalian 18S rRNA [29]. In germinating Schizomyces sp. and human colorectal cancer HCT 116 cells, 18S rRNA contains 2 ac^4^C sites, the first located in helix 34, which helps maintain translation accuracy, and the other in helix 45 [12, 30, 31]. The formed ac^4^C sites in tRNA improve the fidelity of protein translation and maintain heat resistance of organisms [28, 32, 33], and those in rRNA enhance protein translation accuracy.

Most of the early studies focused on defining ac^4^C modifications in tRNAs and rRNAs, and in recent years, research has shifted its focus on ac^4^C in mRNAs [12, 13]. The coding sequence of mRNA near its polyA tail is enriched with ac^4^C residues, and their abundance decreases in the 5′ to 3′ direction corresponding to that of gene transcription. The ac^4^C-enriched mRNAs has a longer half-life. Bioinformatics analysis of ac^4^C peak codon composition indicates that cytidine has a strong enrichment at the wobble sites. The presence of ac^4^C in mRNA coding sequence strongly stimulates translation elongation, while the modification in 5′ UTR regulates translation initiation [15]. Disrupting NAT10 activity ablates ac^4^C at mRNA localization sites, revealing acetylation improves mRNA stability and translation efficiency.

#### The NAT10 enzyme regulates fatty acid metabolism

**Fig. 2:**
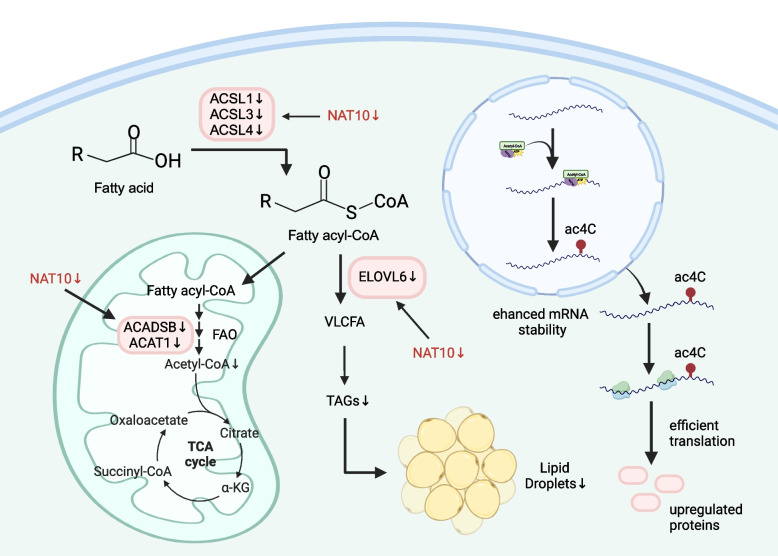
NAT10 involvement in fatty acid metabolism

The proliferation and survival diminish in NAT10-deficient cancer cells [34], prompting further investigations of the underlying mechanism behind this observation. Increasing evidence indicates that fatty acid (FA) metabolism plays an essential role in metastasis and treatment resistance [35–39]. Therefore, exploring the FA metabolism signaling pathway is paramount to studying and treating cancer [40–43]. Moreover, transcriptome profiling has revealed that NAT10 regulates many FA metabolism-related genes: ELOVL fatty acid elongase 6 (*ELOVL6*), acyl-CoA dehydrogenase short/branched chain (*ACADSB*), acetyl-CoA acetyltransferase 1 (*ACAT1*), and acyl-CoA synthetase long chain family member 1, 3, and 4 (*ACSL1*, *3*, and *4*) [34]. In addition, by catalyzing ac^4^C formation, NAT10 improves the mRNA stability of FA metabolism genes, adjusting FA metabolism (Fig. 2). Studying the effect of ac^4^C on palmitate-driven lipid accumulation uncovered that NAT10 regulates palmitate-driven FA metabolism in cancer cells in an ac^4^C-dependent manner. Acetyl-CoA is the substrate of NAT10-catalyzed RNA acetylation and is involved in FA metabolism as the core molecule of the FA metabolism pathway. These findings indicate that NAT10 is involved in lipid accumulation and FA metabolism, underscoring the need to explore its mechanisms in the context of cancer.

Growth and proliferation of cancer cells require biological molecules, including nucleic acids, proteins, and lipids [44, 45]. For example, lipids, such as triacylglycerol, cholesterol, diacylglycerol, and phospholipids, contribute to the energy supply and membrane structural integrity of healthy and cancer cells and act as signaling molecules in various biological processes [46, 47]. Reduced cell growth and proliferation are characteristic of NAT10-depleted cancer cells and could be related to the decrease of the formation of ac4C modification of FA metabolism-related genes caused by NAT10 depletion in these cells,which leads to the reduction of lipid levels. Cell proliferation depends on FA metabolism genes, and exhaustion of NAT10 in cancer cells provokes a dysfunctional FA metabolism, resulting in cell death.

### Technologies for ac^4^C detection

Because the complementary CG base pairs are unaffected by ac^4^C modification, it is undetectable during conventional RNA sequencing. To this end, scientists have developed various ac^4^C RNA detection methods (summarized in Table 1), such as high-performance liquid chromatography (HPLC) and liquid chromatography-mass spectrometry (LC-MS), for qualitative and quantitative detection of ac^4^C RNA. They also developed a high-throughput method called acetylated RNA immunoprecipitation and sequencing (acRIP-seq) that uses specific antibodies against ac^4^C RNA to obtain precise sequence information. N^4^-acetylcytidine sequencing (ac^4^C-seq) is another chemically assisted sequencing method that uses borohydride to reduce ac^4^C to N^4^-acetyl-3,4,5,6-tetrahydrocytidine and determines the ac^4^C RNA sites by detecting the mutation location caused by N^4^-acetyl-3,4,5,6-tetrahydrocytidine in subsequent reverse transcription. Other notable tools predict ac^4^C sites in mRNA, such as PACES and the machine learning model XG-ac^4^C.

**Table 1:** Summary of ac4C detection technologies

Classification	Name	Advantages	Disadvantages	References
HPLC-based methods	RP-HPLC	(1) the main nucleosides and the modified nucleosides obtained by enzymolysis of rna can be separated (2) it does not rely on expensive mass spectrometer detectors	(1) it needs to consume a lot of mobile phase solvent (2) nucleosides with similar retention times could not be qualitatively and quantitatively analyzed	[17, 18]
	UV-HPLC	it can accurately locate the position of the ac4C	(1) the signal cannot be amplified, and the sensitivity is limited (2) limit utility and throughput	[7]
	HPLC conjugated with MISPE	the endogenous pyrimidine nucleosides were selectively extracted from urine, which improved the sensitivity of HPLC analysis results in the subsequent detection process	It is necessary to process the sample under the condition of ph = 10, but AC4c will be hydrolyzed into c under this alkaline condition, and the analysis of AC4c cannot be realized	[21]
	mung bean nuclease cleavage coupled to UV-HPLC	ac4C can be localized to specific sites in cellular rna	it suffers from poor sensitivity due to a lack of signal amplification and requires the synthesis of tiling oligonucleotides, limiting throughput	[20]
HPLC conjugated with MS	LC-MS/HPLC-MS	high precision, high sensitivity, high selectivity	complex operation steps	[13]
Antibody-based methods	acRIP-seq	it can generate thousands of ac4C-enriched transcribed regions	(1) the reads may be biased by the affinity of mRNA and the antibody (2) it cannot provide a base-resolution ac4C map at the transcriptome level	[12, 22]
Borohydride reduction-based methods	borohydridebased reduction	The nucleotide resolution of AC4c can be quantitatively detected	The selectivity is not high, and control tests should be designed in combination with the unstable hydrolysis properties of AC4c in practical applications to further determine the site of AC4c	[7]
	borohydridebased Sanger sequencing	It can sensitively detect a single ac4C site using PCR amplification	it is unable to analyze ac4C in RNAs with densemodified nucleotides	[7]
Computational methods for ac4C site prediction	PACES	good performance	(1) only moieties that are likely to undergo acetylation can be predicted, but the exact location of acetylation cannot be predicted (2) repeated cxx moieties remain ambiguous and the exact form of ac4c acetylation needs to be further studied (3) cross-tissue or cross-species predictions cannot be made due to the limitations of available data	[23]
	XG-ac4C	outperforms the most advanced methods in both cross-validation and independent testing		[24]

#### High-performance liquid chromatography-based methods

High-performance liquid chromatography is an analytical technique used to separate and identify each component in a mixture. Based on the basic principle of column chromatography,it separates the common and modified nucleosides obtained by RNA enzymatic hydrolysis, allowing the detection and analysis of various modified nucleosides in RNA. Reverse HPLC (RP-HPLC) is a separation method based on liquid chromatography technology, which uses the hydrophilic difference of different compounds on reverse fixation to separate, and is a sensitive and effective method for detecting modified nucleosides [48, 49]. It was used in studies with mung bean nuclease protection experiments to identify ac^4^C sites in helix 34 of eukaryotic rRNA, revealing that yeast small nucleolar RNA (snoRNA) is responsible for directing rRNA acetylation [30, 50]. Although HPLC recognizes various modified nucleosides of RNA, it requires a large amount of solvent for separation and does not allow the qualitative and quantitative analysis of nucleosides with similar retention times. In addition, HPLC-based methods cannot amplify the signal, yielding limited sensitivity. In 2008, Damien et al. [51] established molecular imprinted solid phase extraction (MISPE) technology to extract pyrimidines from urine, improving the sensitivity of HPLC results in the subsequent detection. However, because MISPE requires pH = 10 to process the sample, ac^4^C is hydrolyzed to C under these alkaline conditions, hindering ac^4^C analysis.

#### Liquid chromatography-mass spectrometry-based method

LC-MS is a combination of liquid chromatography (LC) and mass spectrometry (MS), which is to analyze the sample by ionizing it in high performance liquid chromatography, separating it according to the mass charge ratio of ions, and then using a mass spectrometry detector to detect the molecular weight information of each ion spectrum peak, so as to achieve the analysis of the sample.Post-translational protein modifications and post-transcriptional non-coding RNA modifications can be studied using mass spectrometry combined with HPLC. For instance, Tardu et al. [13] attempted to quantitatively characterize possible nucleoside variants present in yeast mRNA by an established HPLC tandem mass spectrometry that uses a standard substance to simultaneously measure nucleoside levels with high precision, sensitivity, and selectivity. However, this method is complex and requires a complicated pretreatment of RNA samples.

#### Antibody-based methods

**Fig. 3:**
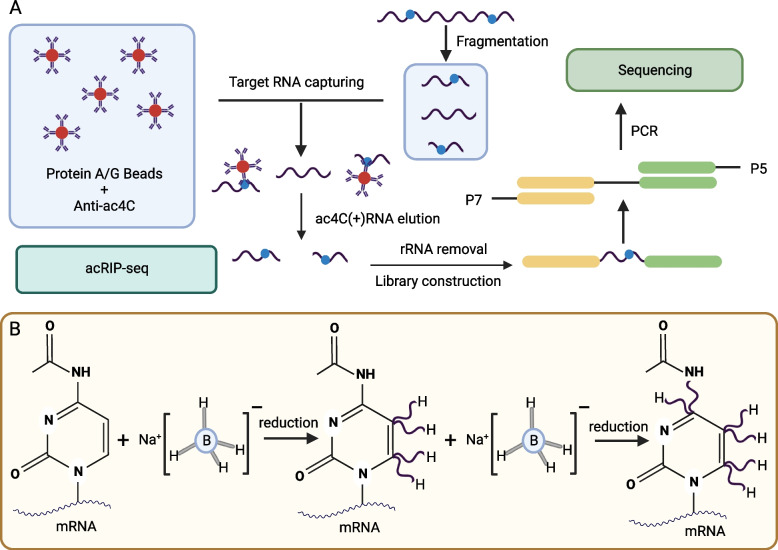
Two methods used to detect ac4C modifications in RNA. **A** Antibody-based method. **B** Borohydride reduction method

Antibody-based methods detect ac^4^C in the RNA sequence using antibodies against ac^4^C to precipitate the modified RNA and identify it with deep sequencing. These techniques have the advantage of signal amplification and have been used to detect ac^4^C in human and viral mRNAs. In 2018, Arango et al. [12] used acetylated RNA immunoprecipitation and sequencing (acRIP-seq) technology to enrich ac^4^C sites in human mRNAs. It the first time to identify these sites in more than 4000 regions and discover their association with the regulation of translation initiation, mRNA localization, and translation inhibition. This technology (Fig. 3A) is based on the principle that antibodies specifically bind ac^4^C residues in the RNA samples. These samples are processed into smaller RNA fragments and mixed with ac^4^C antibody or homologous monoclonal IgG control for immunoprecipitation. Finally, high-throughput sequencing is performed to identify RNA regions that underwent acetylation. Although this method produces thousands ac^4^C-enriched transcription regions, the reading results can be influenced by mRNA and antibody affinity [52].

#### Borohydride reduction-based methods

Since the electron density of the pyridine ring in ac^4^C modification is significantly lower than that of cytidine, ac^4^C is easily reduced by borane. As mentioned previously, ac^4^C is also prone to deacetylation under alkaline conditions. Based on these chemical properties of ac4C, the researchers developed a new detection method called ac4C-seq. In 2018, Thomas et al. [7] used a borane reduction method to detect ac^4^C in 18S rRNA at a single-base resolution. In this method, sodium cyanoborohydride reduces ac^4^C to N^4^-acetyl-3,4,5,6-tetrahydrocytidine under acidic conditions (Fig. 3B). During reverse transcription, the reduced nucleobase is misread as U instead of C, rendering the C to T mutations observable at ac^4^C sites in subsequent cDNA sequencing (ac^4^C-seq). However, the selectivity of this reaction is low since hydrides can also reduce base modifications containing other electron-deficient heteroaromatic rings. Thus, in practical applications, control tests should be designed in combination with the hydrolysis properties of unstable ac^4^C to determine ac^4^C sites with higher accuracy.

The main advantage of ac^4^C-seq over other methods is that it quantitatively detects ac4C at nucleotide resolution and is suitable for detecting ac^4^C reaction kinetics. Its sensitivity depends mainly on the stoichiometric and sequencing depth of ac^4^C, which could theoretically be improved by pre-enriching the samples with ac^4^C-containing RNA using antibodies. However, its foremost limitation is that it entirely depends on the detection of C to T detection, which may underestimate the abundance of modifications on RNA during sequencing.

#### Computational methods for ac^4^C site prediction

Although the human transcriptome has a wide distribution of ac^4^C modification, only a few transcripts with the modified sequences were detected using the previously described methods. Zhao et al. [53] developed a machine learning-based ac^4^C predictor called PACES that helps mine acetylation sequences in human mRNA used as input. It performs prediction at the motif level, extracting sequence features by combining position-specific dinucleotide sequence profile (PSDSP) and K-nucleotide frequency (KNF). Although this method has shown good performance in predicting ac^4^C sites in cross-validation and independent benchmarking tests, it has some limitations. First, because of the limited resolution of the high-throughput tests to identify ac^4^C modification, PACES only predicts the motifs where acetylation is likely to occur, not the exact location. Second, the repetitive CXX motif remains obscure. Third, with limited available data, PACES cannot predict across different tissues or species.

Alam et al. [54] proposed a computational model called XG-ac^4^C for determining ac^4^C modification sites in mRNA based on the XGboost algorithm. The evaluation of the XG-ac^4^C model showed that it outperforms the most advanced methods in cross-validation and independent testing.

### Roles of ac^4^C in cancer

Cancer is a serious disease threatening human health, and its progression is related to the formation of ac^4^C modification in RNA. The NAT10 writer protein is highly expressed in tumor tissues, catalyzing the formation of ac^4^C residues in RNAs encoded by various cancer-related genes and contributing to tumor evolution (summarized in Table 2). Thus, future studies about the relevant affected by ac^4^C modification should reveal its mechanisms in tumor evolution to provide new ideas and methods for early tumor diagnosis and treatment. Ultimately, novel treatment strategies may improve the survival and life quality of patients with cancer.

**Table 2:** Functions of oncogenes modified by ac4C in cancers

Type	Factor	Tumor	Pathway	Targets	expression	Functions	References
Writer	NAT10	Colon cancer	NAT10-FSP1-ferroptosis signaling	FSP1	overexpression	proliferation, metastasis	[55]
		Cervical cancer	NAT10-ac4C-HNRNPUL1 axis	HNRNPUL1	overexpression	tumorigenicity, migration, invasion	[56]
		Gastric cancer	the Hp-NAT10-MDM2-p53 axis	MDM2	overexpression	proliferation	[57]
				COL5A1	overexpression	metastasis, EMT	[58]
		Pancreatic cancer	LINC00623/NAT10 signaling axis	MUC4、LAMB3、PHGDH	overexpression	tumorigenicity, migration	[59]
			TGF-β pathway	TGF-β	overexpression	metastasis	[60]
			PI3K-AKT pathway	AKT	overexpression	proliferation	[60]
		Bladder cancer		BCL9L	overexpression	invasion, metastasis, proliferation	[33]
				SOX4	overexpression	invasion, metastasis, prognosis	[33]
				AKT1	overexpression	invasion	[33]
		Esophageal cancer	the NAT10-ac4C-tRNA-EGFR oncogenic axis	EGFR	overexpression	tumorigenicity	[61]
				CTC-490G23.2	overexpression	invasion, metastasis	[62]
		Hepatocellular carcinoma		COL15A1	overexpression	prognosis	[63]
				G6PD、TP53I3	overexpression	prognosis	[63]
		Triple receptor-negative breast cancer		SNHG14 (H3K27)	overexpression	trastuzumab resistance	[64]
				GHSROS	overexpression	proliferation, migration	[64]
				NONHSAT101069	overexpression	epirubicin resistance , migration, invasion	[64]
				RP11-22 N19.2	overexpression	prognosis	[64]
				USP8, COL3A1, TRIR	overexpression	prognosis	[64]
Reader	Unknown						
Eraser	Unknown						

#### Colon cancer

Colon cancer is a common malignant tumor of the digestive tract and one of the leading causes of cancer-related deaths worldwide [65]. Relapse, metastasis, or death occurs in 30 to 50% of patients with this disease within 5 years of treatment [66, 67]. Although screening and treatment strategies for colon cancer have improved in recent years, the outcome for patients with advanced colon cancer remains poor mainly because the molecular mechanisms behind the disease development are still elusive [55, 68–70]. Therefore, studying these mechanisms is necessary to find novel strategies that predict colon cancer occurrence or effective targeted therapy that improves patient prognosis.

Zheng et al. [71] established NAT10 knockdown and overexpression cell lines by lentiviral transduction to study the role of NAT10 in colon cancer. They found that NAT10 levels significantly correlate with cell proliferation; knocking down the protein significantly inhibits cell proliferation. Other studies have shown that NAT10 promotes the proliferation of colon cancer cells, and the mechanism that enhances the progression of colon cancer cells is active in the G0/G1 to G2/M phase of the cell cycle. In addition, NAT10 is involved in colon cancer cell metastasis; down-regulating NAT10 inhibits cell migration and reduces the number of aggressive cancer cells. In summary, NAT10 expression is up-regulated in colon cancer and is associated with poor prognosis.

In order to further explore the potebtial molecular mechaniam of NAT10 promoting proliferation and metastasis of colon cancer cells, Zheng et al. [71] conducted transcriptomic profiling experiments, showing that ferroptosis suppressor protein 1 (FSP1) is deregulated in NAT10 knockdown cells. Dalhat et al. [56] explored the relationship between NAT10 and FSP1 proteins and discovered that NAT10 acts as an epigenetic transcriptome regulator of the ferroptosis pathway in cancer cells via FSP1. Real-time PCR and Western blotting confirmed that FSP1 expression increases in NAT10 overexpression cells but decreases in NAT10 knockdown cells. Moreover, the levels of ac^4^C-acetylated *FSP1* mRNA continuously drop in NAT10 knockdown cells but rise in the overexpression cells, agreeing with the protein expression. To summarize, NAT10 catalyzes ac^4^C formation in *FSP1* mRNA to enhance its stability in colon cancer cells, increasing FSP1 expression. The FSP1 protein is a glutathione-independent ferroptosis inhibitor. The NAT10 knockdown cells also exhibit enhanced GSH consumption and increased lipid reactive oxygen species, ferrous iron, and malondialdehyde levels. In addition, transmission electron microscopy of these cells showed that the mitochondrial matrix condenses and forms enlarged cristae, suggesting knocking down NAT10 induces ferroptosis in colon cancer cells. Ferrostatin-1 is a synthetic inhibitor that represses lipid peroxidation necessary to prime ferroptosis. Ferrostatin-1-suppressed ferroptosis does not affect the proliferation and metastasis of carrier-controlled cells but increases both processes in NAT10 knockdown cells.

**Fig. 4:**
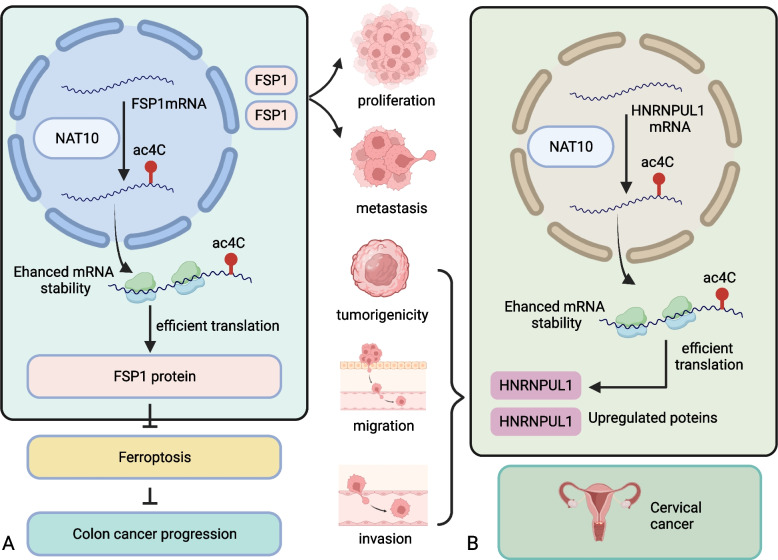
NAT10-related mechanisms involved in tumor progression. **A** NAT10-mediated ac4C in FSP1 mRNA participates in colon cancer progression. **B** NAT10-mediated ac4C in HNRNPUL1 mRNA participates in cervical cancer progression

These studies suggest that inhibiting ferroptosis reverses the inhibition of cell proliferation and metastasis mediated by NAT10 down-regulation in colon cancer cells. In summary, NAT10 improves the stability of *FSP1* mRNA to enhance FSP1 expression by catalyzing ac^4^C formation in *FSP1* mRNA. The enhanced FSP1 expression, in turn, inhibits the ferroptosis of colon cancer cells, promoting their proliferation and metastasis (Fig. 4A).

#### Cervical cancer

Cervical cancer is the most common malignant neoplasm of the reproductive tract, with the highest morbidity and mortality among women [72]. In recent years, the incidence of this disease has increased in younger women, and HPV infection is its most important risk factor [73, 74]. Interestingly, not all patients with HPV develop cervical cancer, suggesting that HPV infection is insufficient to develop the disease [75]. Indeed, epigenetic events could be necessary for the disease onset. The incidence and mortality of cervical cancer have decreased significantly in recent decades, possibly because of the widespread use of cervical cancer cytological screening that allows early detection and treatment [57]. However, because various treatments (surgery, radiotherapy, chemotherapy, immunotherapy, etc.) have low clinical effects in patients with advanced or recurrent cervical cancer, the prognosis for these patients remains poor. Therefore, studying the molecular mechanisms of cervical cancer and finding new biomarkers is crucial to improving patient survival.

Transcriptome analysis by Long et al. [75] revealed that NAT10 expression is upregulated in cervical cancer cells, and knocking out NAT10 inhibits their proliferation, invasion, and migration. Xenotransplantation models confirmed the carcinogenic function of NAT10. Recent evidence shows that the target of NAT10 in cervical cancer is heterogeneous nuclear ribonucleoprotein U like 1 (HNRNPUL1) [75]. The NAT10 protein regulates HNRNPUL1 expression in cervical cancer cells by catalyzing ac^4^C formation and increasing the stability of *HNRNPUL1* mRNA. In conclusion, NAT10 enhances the stability of *HNRNPUL1* mRNA through ac^4^C modification, promoting the development of cervical cancer (Fig. 4B).

A database analysis was conducted to study the relationship between NAT10 and the malignant behavior of cervical cancer cells. It found that high NAT10 expression is related to poor prognosis of patients with cervical cancer. Indeed, overexpressing NAT10 in these cells improves their growth and proliferation and promotes invasion and migration. Bioinformatic analysis showed that discoidin protein domain receptor tyrosine kinase 1 (DDR1) may be the downstream target gene of NAT10. Western blotting and RT-qPCR showed that NAT10 overexpression significantly upregulates DDR1 protein and mRNA levels, suggesting NAT10 increases DDR1 expression by catalyzing ac^4^C in *DDR1* mRNA. Therefore, NAT10 improves the stability of *DDR1* mRNA by acetylation, promoting the growth, proliferation, invasion, and migration of cervical cancer cells.

#### Gastric cancer

Gastric cancer (GC) is a common malignant tumor of the digestive tract and the fourth leading cause of cancer-related deaths globally [76]. The disease is typically caused by an infection with *Helicobacter pylori* [58]. Patients with early gastric cancer show excellent improvement after surgical treatment and adjuvant therapy, with a 5-year survival rate of over 90%. However, those with the advanced disease still have a poor prognosis, with the 5-year survival rate less than 25% [77]. Therefore, understanding the underlying mechanisms and developing new targeted treatment options is necessary to improve the clinical prognosis of patients with GC.

**Fig. 5:**
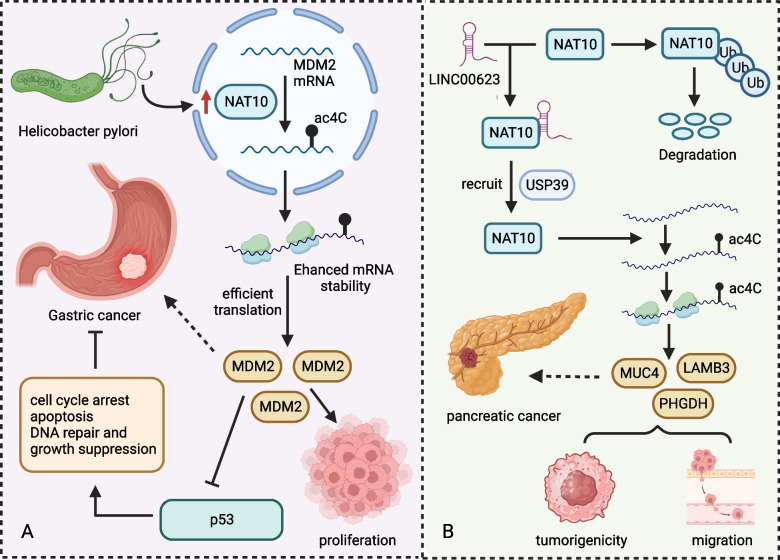
NAT10-associated pathways related to tumor development. **A** The HP-NAT10-MDM2-p53 signaling axis promoting GC development. **B** The LINC00623 lncRNA-NAT10 signaling axis promoting pancreatic cancer development

Deng et al. [78] studied the relationship between ac4C modification and GC occurrence and found that NAT10 is significantly up-regulated in GC cells versus healthy cells. *H. pylori* infection contributes to NAT10 induction, regulating p53 stability via MDM2 proto-oncogene (MDM2). The NAT10 enzyme catalyzes ac^4^C formation in *MDM2* mRNA, improving its stability and overexpression. Enhanced MDM2 expression degrades p53, promoting GC occurrence (Fig. 5A). Knocking out NAT10 in human gastric cancer AGS cells reduces ac^4^C modification of total RNA and mRNA and represses proliferation and invasion of tumor cells. In addition, the cells undergo a considerable G2/M cell cycle arrest and apoptosis. These NAT10 effects are reversed when treating the cells with the NAT10 inhibitor Remodelin [79], demonstrating its anti-GC activity.

The overexpression of NAT10 in GC also up-regulates collagen type V alpha 1 chain (COL5A1) through ac^4^C formation, promoting GC epithelial-mesenchymal transition (EMT) and metastasis. Zhang et al. [80] conducted in vivo and in vitro tests to investigate the relationship between NAT10 and GC malignant transformation. They showed that NAT10 down-regulation inhibits while NAT10 overexpression enhances GC cell migration. They also confirmed that NAT10 promotes EMT progression in gastric cancer cells using Western blotting, RT-qPCR, and immunofluorescence.

#### Pancreatic cancer

Pancreatic cancer is highly malignant and is the seventh leading cause of cancer death worldwide [81]. While treatment has significantly increased the survival of a small number of patients with this disease over the last decades, the 5-year survival rate for pancreatic cancer remains high [59, 60], and its incidence is expected to surpass that of breast cancer in the upcoming years [82]. Treating this cancer comes with many challenges that arise from late diagnosis and treatment resistance. Although surgery has the potential to cure pancreatic cancer, it usually requires a combined treatment intervention whose adverse reactions often seriously affect the life quality of patients [83]. Therefore, developing new diagnostic methods and therapeutic strategies with high specificity and sensitivity is the priority of current research efforts.

Xu et al. [84] collected genomic data from pancreatic cancer tissues and constructed NAT10 subgroup phenotypes to assess the association between NAT10 levels and clinicopathological characteristics of patients with pancreatic cancer. This group discovered that NAT10 is involved in the clinical outcome of the disease and tumor tissue infiltration, drug resistance, migration, and clonogenic potential. They showed that pancreatic cancer tissues exhibit abnormally increased NAT10 expression, and the patients with the abnormal expression had a poor prognosis. Moreover, they also showed AKT serine/threonine kinase (AKT) activation in cancer tissues positively correlates with cell proliferation. Thus, abnormal NAT10 expression may promote malignant proliferation of pancreatic cancer by triggering the PI3K-AKT pathway. The aberrant NAT10 expression also promotes TGF-β signaling and angiogenesis, implying it promotes metastasis of pancreatic cancer cells. Indeed, knocking down NAT10 with two siRNAs reduces colony-forming and migration ability of pancreatic cancer cells. Gemcitabine is the first-line treatment for unresectable locally advanced or metastatic pancreatic cancer. Xu et al. [84] found that high NAT10 expression increases the resistance of pancreatic cancer cells to gemcitabine therapy. Through the study, they concluded that NAT10 promotes tumor cell migration and gemcitabine resistance by mediating the formation of ac4C modification in mRNA.In conclusion, NAT10 down-regulation inhibits the migration and clonogenic capacity of pancreatic cancer cells and reduces their resistance to gemcitabine,which could be a potential target for pancreatic cancer treatment.

Feng et al. [85] identified a new long non-coding RNA (lncRNA) *LINC00623* and confirmed its diagnostic value in patients with pancreatic cancer. They showed that *LINC00623* lncRNA promotes tumorigenicity and migration of pancreatic cancer cells in vivo and in vitro. Mechanically, it is up-regulated in tumor tissues and binds to NAT10, recruiting ubiquitin specific peptidase 39 (USP39) to block NAT10 ubiquitination-dependent degradation,thus promoting the formation of ac4C modification in mRNA, enhancing gene expression and leading to tumor occurrence (Fig. 5B). The team also found that the inhibitor ASO-LINC00623 represses *LINC00623* lncRNA expression and significantly reduces the proliferation and EMT of pancreatic cancer cells, revealing a great potential for pancreatic cancer treatment. Together, these data suggest that *LINC00623* lncRNA is a potential therapeutic target for pancreatic cancer.

#### Bladder cancer

Bladder cancer (BLCA) is a frequent malignant tumor of the urinary system. Approximately 550,000 people worldwide are diagnosed with this cancer yearly, making it the 12th most common malignant neoplasm [86]. The incidence of BLCA increases every year, and treating the recurrent and advanced disease after complete resection remains a daunting challenge [87]. Therefore, identifying biological markers and effective treatment strategies are urgently needed to combat the disease.

Healthy tissues have considerably lower NAT10 expression than BLCA tissues. In addition, NAT10 levels positively correlate with tumor aggressiveness, and patients with lymph node metastasis have elevated NAT10 protein. The overall survival rate of patients with low NAT10 expression is higher than in patients with high expression, suggesting that NAT10 expression predicts BLCA prognosis. Antagonistic NAT10 and NAT10 gene knockout methods delay BLCA progression, and targeting NAT10 may be a new strategy for BLCA treatment. In addition, high NAT10 expression in BLCA tissues could be used as a predictor of poor prognosis.

Wang et al. [29] uncovered that NAT10 is highly expressed in BLCA tissues and is promotes tumor proliferation and migration by catalyzing ac^4^C modification in target transcripts. The research group used transcriptome-wide acRIP-seq to study NAT10 localization and ac^4^C-modified downstream genes in specific mods. It identified that NAT10 directly binds 3 ac^4^C-enriched targets ac^4^C: BCL9 like (BCL9L), SRY-box transcription factor 4 (SOX4), and AKT1. The expression of these targets is significantly reduced after knocking out NAT10, indicating it enhances their expression. Furthermore, NAT10 promotes proliferation and tumorigenesis in vivo by acetylating downstream targets. For instance, BCL9L positively correlates with tumor invasiveness, migration ability, cell polymorphism, and tumor progression. The survival time of patients with low BCL9L expression is significantly higher than that of patients with high expression, suggesting this gene may be used as a prognostic indicator of BLCA. Likewise, SOX4 expression positively correlates with tumor invasion ability, and patients with high SOX4 expression are more likely to develop lymph node metastasis. Patients with early-stage BLCA also have lower SOX4 levels than those with advance-stage, suggesting that high SOX4 expression is closely related to poor prognosis. The expression of AKT1 is also related to tumor invasion and migration.

#### Esophageal cancer

Esophageal cancer is a highly aggressive malignant tumor. It causes 400,000 deaths yearly and is the eighth most common cancer worldwide [88]. Patients with this disease have high mortality and poor prognosis [61, 62]. Recent clinical trials have shown that patients with advanced disease benefit from gefitinib treatment, but its response rate is low [89, 90]. As demonstrated for other cancers in this review, studying the molecular mechanisms of esophageal cancer development and drug resistance and formulating effective treatment strategies is paramount.

Yu et al. [91] used a method that analyzes ac^4^C sites at nucleotide resolution to determine whether lncRNAs contain ac^4^C modification. The team found that NAT10 catalyzes ac^4^C formation in *CTC-490G23.2* lncRNA, inducing transcript expression in primary esophageal cancer and metastatic tissues. They also demonstrated that *CTC-490G23.2* lncRNA is associated with invasion and metastasis of tumor cells. Mechanistically, this transcript promotes the binding of *CD44* precursor mRNA to polypyrimidine tract-binding protein 1 (PTBP1), shifting the splicing of the precursor mRNA from the common to the oncogenic variant isoform (*CD44s*-*CD44v*). Consequently, the oncogenic isoform *CD44v* binds to vimentin and increases its stability, suggesting that *CTC-490G23.2* lncRNA overexpression stimulates EMT in cancer cells. Because high expression of *CTC-490G23.2* lncRNA and *CD44v* mRNA correlates with poor prognosis, these transcripts could be prognostic biomarkers of esophageal cancer. Moreover, targeting *CTC-490G23.2* lncRNA with antisense oligonucleotides (ASOs) inhibits cancer metastasis significantly. These findings will further our understanding of lncRNA ac^4^C modification and provide effective treatment strategies for developing new therapeutic approaches.

Wei et al. [92] performed several studies to confirm that NAT10 participates in the occurrence and development of esophageal cancer. This group showed that NAT10 is overexpressed in esophageal cancer tissues and is associated with disease prognosis. In addition, depleting NAT10 reduces the pool of ac^4^C-modified tRNAs essential for optimal mRNA translation efficiency. The group further identified that the epidermal growth factor receptor (EGFR) protein is a downstream target of NAT10, promoting its carcinogenic function. Gefitinib is an EGFR inhibitor that can improve the survival rate of patients with advanced esophageal cancer. However, its widespread clinical application as a second-line treatment has been hindered by drug resistance [89, 93]. Wei et al. found that NAT10 promotes the resistance of esophageal cancer cells to gefitinib therapy. Remarkably, NAT10 depletion and gefitinib therapy synergistically inhibit cancer cell invasion and migration, showing that this approach alleviates gefitinib resistance and provides novel insights for developing effective cancer treatment strategies.

#### Hepatocellular carcinoma

Liver cancer encompasses hepatocellular carcinoma (HCC) and cholangiocarcinoma, with high morbidity and mortality. Its most represented subtype is HCC, accounting for 90% of primary liver cancer cases [94, 95]. In China, patients with HCC have a 5-year survival rate of only 12% [63] and develop the disease owing to various risk factors, especially hepatitis virus infection [96, 97]. The first-line treatment against HCC consists of radiotherapy, chemotherapy, surgical resection, and liver transplantation [98]. However, because the disease has complex pathogenesis, rapid proliferation, extensive invasion, and migration, patients have limited benefit from the treatment and are prone to relapse and metastasis, conferring poor prognosis. Therefore, discovering new HCC biomarkers for early diagnosis and exploring the molecular mechanisms of HCC occurrence and development is needed to improve cancer prognosis.

Liu et al. [99] established an ac^4^C score model to study the role of ac^4^C mRNA modification in HCC development and progression. The research team identified differentially expressed genes between tumor and healthy tissues and selected 3 genes to construct a risk model (ac^4^C score): collagen type XV alpha 1 chain (COL15A1), glucose-6-phosphate dehydrogenase (G6PD), and tumor protein p53 inducible protein 3 (TP53I3). The model classified the patients into 2 groups with different prognoses. The team also used bioinformatics tools to confirm the relationship between the ac^4^C score and tumor stemness or tumor microenvironment infiltration, indicating that the ac^4^C score is a suitable biomarker to predict the prognosis of patients with HCC.

#### Triple receptor-negative breast cancer

Breast cancer is the most common malignant neoplasm in women [100, 101], and its incidence in China has been increasing significantly over the past years [102]. Triple receptor-negative breast cancer (TNBC) does not express human epidermal growth factor receptor 2 (HER2), estrogen, and progesterone receptors [64, 103], contributing to its high malignancy and impeding treatment efforts. Tumor patients have short life span, high early recurrence rate and poor prognosis [104, 105]. TNBC does not benefit from endocrine therapy for breast cancer as well as anti-HER2-targeted therapy.

Zhang et al. [106] collected TNBC tissue samples and classified them according to NAT10 expression to understand the role of ac^4^C modification in TNBC progression and help design new personalized treatment plans and prognostic assessment. They found 703 lncRNAs differentially expressed between the high and low NAT10 expression groups, of which 20 lncRNAs were associated with disease prognosis. The results suggest that NAT10 regulates lncRNA expression via ac^4^C modification, affecting TNBC prognosis. Ultimately, understanding the mechanisms of lncRNAs will help us predict drug targets and drug sensitivity of TNBC cells.

## Conclusion

**Fig. 6:**
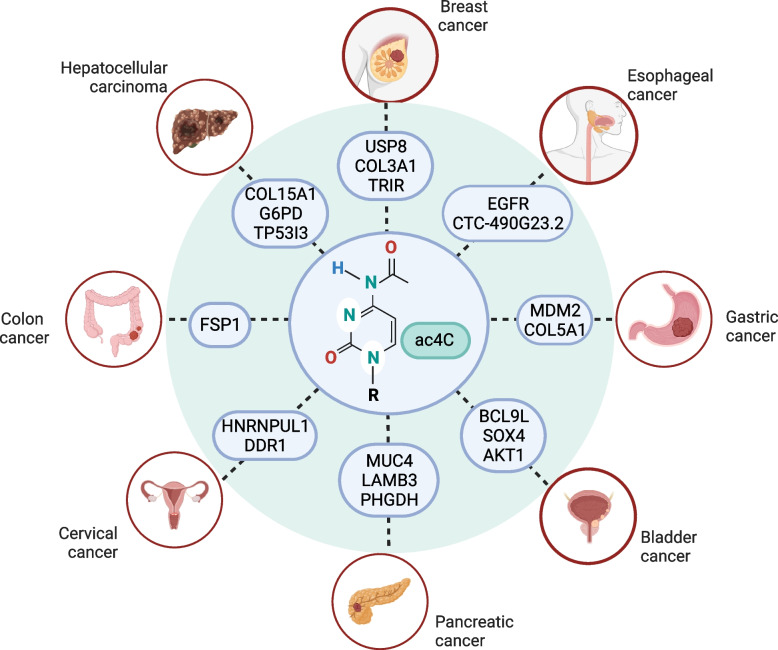
Diagram summarizing cancers covered in the review and their associated ac4C-modified oncogenes

The NAT10 protein is the first enzyme shown to catalyze the formation of acetylation in RNAs. Although research on NAT10 is limited, it revealed this enzyme has a key role in the development of various malignant tumors. It is involved in the progression of multiple tumors by catalyzing the formation of ac^4^C modifications in many RNA species (Fig. 6) and is overexpressed in many cancers. In colon cancer cells, FSP1 expression is upregulated by acetylation modification of FSP1, inhibiting ferroptosis but promoting metastasis and proliferation. In pancreatic cancer, NAT10 overexpression promotes malignant cell proliferation by activating the PI3K-AKT pathway. A newly discovered *LINC00623* lncRNA, which binds to NAT10, promotes the recruitment of the deubiquitinating enzyme USP39 and reduces the ubiquitination-dependent NAT10 degradation. The *LINC00623* lncRNA inhibitor (ASO-LINC00623) significantly reduces tumor burden. In BLCA, NAT10 overexpression acetylates BCL9L, SOX4, and AKT1, promoting tumor invasion and metastasis. In GC, NAT10 overexpression up-regulates *MDM2* and *COL5A1* mRNA expression through acetylation, promoting GC occurrence and metastasis. In esophageal cancer, NAT10 overexpression is further up-regulated in primary cancer and metastatic tissues through CTC-490G23.2 lncRNA acetylation, promoting tumor invasion and metastasis. In HCC and TNBC, NAT10 is up-regulated, and its detailed mechanism in these cancers demands further research. In cervical cancer, NAT10 overexpression induces *HNRNPUL1* and *DDR1* mRNA expression by promoting acetylation, enhancing tumor proliferation, invasion, and metastasis. In addition, NAT10 has aberrant expression in other malignant tumors undiscussed, such as melanoma, epithelial ovarian, and non-small cell lung cancers. However, we did not discuss its biological functions and roles in these cancers as they are unknown and require further exploration.

ac4C modification is a newly discovered RNA modification, and its research is still incomplete. Only one writer protein has been found so far. Whether other writers, readers and erasers exist remains to be investigated. The specific mechanism of ac4C modification in RNA catalyzed by NAT10 to participate in tumor development is still unclear. Secondly, some studies have found that Remodelin has anticancer activity in some cancers, and may be used as a new target for cancer therapy. Fatty acid metabolism plays a crucial role in lipid accumulation, and lipid substances are essential for energy supply and membrane structural integrity. Previous studies have shown that NAT10 can mediate ac4C modification of key genes of fatty acid metabolism, regulate gene expression, and affect lipid formation. However, no studies have proven the correlation between this pathway and cancer progression, which can be used as a direction for further research.

## Data Availability

Not applicable.

## Abbreviations

NAT10: N-acetyltransferase 10
ac4C: N^4^-acetylcytidine
m5C: 5-methylcytosine
m1A: N^1^-methyladenosine
m6A: N^6^-methyladenosine
m7G: 7-methylguanosine
ψ: pseudouracil
ac4Cm: N^4^-acetyl-2′-O-methylcytidine
ac6C: N^6^-acetyladenosine
HPLC: High-performance liquid chromatography
LC-MS: Liquid chromatography-mass spectrometry
acRIP-seq: Acetylated RNA immunoprecipitation and sequencing
ac4C-seq: N4-acetylcytidine sequencing
RP-HPLC: Reverse HPLC
snoRNA: small nucleolar RNA
MISPE: Molecular imprinted solid phase extraction
PSDS: Position-specific dinucleotide sequence profile
KNF: K-nucleotide frequency
GNAT: G protein subunit alpha transducin
FA: Fatty acid
ELOVL6: ELOVL fatty acid elongase 6
ACADSB: Acyl-CoA dehydrogenase short/branched chain
ACAT1: Acetyl-CoA acetyltransferase 1
ACSL1: Acyl-CoA synthetase long chain family member 1
ACSL3: Acyl-CoA synthetase long chain family member 3
ACSL4: Acyl-CoA synthetase long chain family member 4
FSP1: Ferroptosis suppressor protein 1
HNRNPUL1: Heterogeneous nuclear ribonucleoprotein U like 1
DDR1: Discoidin protein domain receptor tyrosine kinase 1
GC: Gastric cancer
MDM2: MDM2 proto-oncogene
COL5A1: Collagen type V alpha 1 chain
EMT: Epithelial-mesenchymal transition
AKT: AKT serine/threonine kinase
lncRNA: Long non-coding RNA
USP39: Ubiquitin specific peptidase 39
BLCA: Bladder cancer
BCL9L: BCL9 like
SOX4: SRY-box transcription factor 4
PTBP1: Polypyrimidine tract-binding protein 1
ASOs: Antisense oligonucleotides
EGFR: Epidermal growth factor receptor
HCC: Hepatocellular carcinoma
COL15A1: Collagen type XV alpha 1 chain
G6PD: Glucose-6-phosphate dehydrogenase
TP53I3: Tumor protein p53 inducible protein 3
TNBC: Triple receptor-negative breast cancer
HER2: Human epidermal growth factor receptor 2
ASO-LINC00623: LINC00623 lncRNA inhibitor
